# Antioxidant Therapy against Oxidative Damage of the Inner Ear: Protection and Preconditioning

**DOI:** 10.3390/antiox9111076

**Published:** 2020-11-02

**Authors:** Jhang Ho Pak, Yehree Kim, Junyeong Yi, Jong Woo Chung

**Affiliations:** 1 Asan Medical Center, Department of Convergence Medicine, College of Medicine, University of Ulsan and Asan Institute for Life Sciences, Seoul 05505, Korea; jhpak@amc.seoul.kr; 2 Asan Medical Center, Department of Otorhinolaryngology—Head and Neck Surgery, College of Medicine, University of Ulsan, Seoul 05505, Korea; yehreek@wmail.ulsan.ac.kr (Y.K.); junyi@wmail.ulsan.ac.kr (J.Y.)

**Keywords:** reactive oxygen species, antioxidants, inner ear, preconditioning, hair cells

## Abstract

Oxidative stress is an important mechanism underlying cellular damage of the inner ear, resulting in hearing loss. In order to prevent hearing loss, several types of antioxidants have been investigated; several experiments have shown their ability to effectively prevent noise-induced hearing loss, age-related hearing loss, and ototoxicity in animal models. Exogenous antioxidants has been used as single therapeutic agents or in combination. Antioxidant therapy is generally administered before the production of reactive oxygen species. However, post-exposure treatment could also be effective. Preconditioning refers to the phenomenon of pre-inducing a preventative pathway by subtle stimuli that do not cause permanent damage in the inner ear. This renders the inner ear more resistant to actual stimuli that cause permanent hearing damage. The preconditioning mechanism is also related to the induction of antioxidant enzymes. In this review, we discuss the mechanisms underlying antioxidant-associated therapeutic effects and preconditioning in the inner ear.

## 1. Oxidative Damage in the Inner Ear

In addition to hearing, distinguishing sounds is also a key function of the inner ear. However, the inner ear may sometimes be slowly damaged from birth. Typical damage of the normal function of the inner ear is caused by noise exposure, ototoxic drugs, aging, and autoimmune damage; sometimes, sudden loss of inner ear function may also be caused by unknown reasons. The incidence of impaired hearing is currently increasing [1]. Hearing loss among adolescents is also increasing, which represents a possible burden for future societies [2]. Among adolescents, hearing loss affects both hearing itself and the central auditory processing involved in recognizing and reacting to sounds, therefore, interfering with their social life [3].

### 1.1. Noise-Induced Hearing Loss

The major mechanisms of inner ear damage induced by noise (noise-induced hearing loss) are physical damage [4], decreased blood flow and hypoxia [5,6,7], glutamate-associated excitotoxicity of the inner hair cell synaptic area [8], and tissue damage caused by free radicals [9,10]. In particular, several studies have reported noise-dependent generation of reactive oxygen species (ROS) in the inner ear by various mechanisms. The production of free radicals has been proven by the detection of ROS, an increase in the levels of their metabolites [10,11,12,13], and the role of antioxidants in the prevention of hearing loss [14,15,16]. For example, Ohinata et al. used 8-isoprostane, a lipid peroxidation product, as an indicator of ROS generation [16]. Pigmented guinea pigs were exposed to noise and the amount of 8-isoprostane was measured over time. Notably, the amount of 8-isoprostane in the inner ear progressively increased during noise exposure, while decreasing at the end of the experiment. Subsequently, immunohistological analysis mainly highlighted active immunostaining at the cochlear second turn, especially on outer hair cells. These results show that noise exposure can trigger ROS generation, which is related to internal tissue damage. Noise also reportedly increases the level of reactive nitrogen species (RNS) in the inner ear [17]. The outcome of ROS damage is usually apoptosis [18].

One of the factors that intensifies noise-induced hearing loss is smoking. Smoking directly supplies exogenous ROS and also promotes the generation of endogenous ROS, thereby increasing ROS concentration in tissues. In an animal model, smoking before noise exposure aggravated noise-induced hearing loss and induced a permanent threshold shift, even at the noise level that normally resulted in a temporary threshold shift [19]. 

### 1.2. Age-Related Hearing Loss

Age-related hearing loss or presbycusis is a phenomenon caused by the loss of hair cells and spiral ganglion cells in the cochlea. Studies have shown that environmental and genetic factors were involved in the degeneration of cochlear hair cells [20]. Moreover, molecular analyses have indicated that multiple cell signaling mechanisms affected pathological changes that caused hearing loss in aged mice [4].

ROS play an essential role in such age-related hearing loss. Indeed, ROS cause genetic and cell mutations inhibiting cellular functioning, and consequently play an important role in senescence or age-related deafness [21]. With aging, ROS accumulate in the body, substantially reduces blood flow in the circulatory system, especially in the inner ear. Such prolonged, aging-associated reduced blood flow causes excessive production of free radicals, resulting in mitochondrial DNA destruction and damage of the inner ear structure [17,22,23]. Consistently, in patients with mutations in the N-acetyl transferase (*NAT*) gene, which is involved in the metabolism and detoxification of free radical species, age-related hearing loss occurs more frequently than in healthy subjects [24].

Previous studies have also reported the association between metabolic syndrome and ROS production. It is generally acknowledged that ROS affect tissue integrity, and thus the progression of metabolic syndrome. In particular, excessive salt consumption has been reported to be a relevant factor in this process [25]. Metabolic syndrome has also been found to be related to age-related hearing loss. Specifically, Kim et al. measured the abdominal fat composition of several human subjects by computed tomography (CT), analyzed its association with hearing, and reported a positive correlation between visceral accumulation of adipose tissue and impaired hearing ability [26]. 

In addition, in a longitudinal study, which recruited 1381 people, hearing loss was found to be more prominent in women over 50 years of age affected by metabolic syndrome [27]. Elevated visceral adipose tissue levels may induce hearing loss because they cause the activation of adipocytokine-related inflammatory processes and oxidative stress. Furthermore, the amount of circulating adiponectin, a hormone associated with metabolic syndrome that exerts anti-oxidative activity involving glucose metabolism and fatty acid oxidation, is associated with hearing loss [28]; low plasma adiponectin affects age-related hearing loss, especially in women over 55 years of age [29,30,31]. In conclusion, metabolic syndrome is associated with hearing loss through oxidative stress.

### 1.3. Ototoxicity

Aminoglycoside antibiotics and cisplatin are known to cause ototoxic hearing loss. In particular, cisplatin causes excessive free radical production in cells [32], resulting in their accumulation due to the lack of an adequate concentration of glutathione (GSH) [33] or the inactivation of the antioxidant system [34,35]. Therefore, cisplatin-induced apoptosis may occur through the activation of the mitochondrial pathway [36] and cell death inhibition could be a treatment strategy for cisplatin-related ototoxicity, as suggested by multiple reports. Other studies have reported that aminoglycoside antibiotics, such as gentamicin, activated ROS production in the inner ear, and causing damage [37]. However, several studies have also shown that gentamicin-related ototoxicity can be inhibited and prevented [38].

### 1.4. Sudden Hearing Loss and Immune-Mediated Hearing Loss

Sudden hearing loss is a sensorineural process that occurs suddenly, within hours or from two to three days, without a well-known cause. As the treatment prognosis varies, the causes are thought to be multivariate, including viral infections, vascular disorders, and autoimmune diseases [39,40,41,42,43]. Changes in the levels of cytokines, such as tumor necrosis factor alpha (TNF-α) and interleukin-6 (IL-6), are correlated with the prognosis in patients with sudden hearing loss [44]. Notably, these cytokines are associated with ROS damage and with the generation and progression of ROS-related inner ear damage.

Immune-mediated hearing loss is a rare condition associated with autoimmune problems, characterized by frequent, recurrent, and chronic progress, which is known to account for approximately 1% of all hearing loss cases or hearing loss in approximately five of every 100,000 people per year [42]. However, in more common diseases, such as sudden hearing loss and Ménière’s disease, autoimmune reactions are also considered to have a higher incidence than that currently known [40,44,45].

Sudden hearing loss and autoimmune hearing loss have a common factor, i.e., they respond to steroid treatment when associated with rapid hearing loss. The use of steroids or immunosuppressants has been attempted to control the process of immune-related hearing loss. 

TNF-α is a pro-inflammatory cytokine that contributes to the maintenance of cellular homeostasis. TNF-α is rarely observed in normal internal organs; however, its levels increase with age [46], inflammation [47], exposure to noise [48], and exposure to ototoxic drugs [49]. Therefore, it can be inferred that TNF-α levels increase in the inner ear, possibly causing hearing loss. 

For vestibular schwannoma-related sensorineural hearing loss, in particular, the TNF-α level within vestibular schwannoma secretions have been reported to be associated with a degree of hearing loss [41]. In fact, distortion product otoacoustic emissions did not change when TNF-α was perfused intracochlearly, but the amplitude of the compound action potential became smaller and the inner hair cells showed synaptic degeneration [21]. As such, TNF-α appears to affect hearing through internal changes.

Anti-TNF-α therapy was studied to prevent such damage. For example, recent studies have reported improved hearing upon intratympanic injection of infliximab, a TNF-α inhibitor, in refractory and recurrent patients with hearing loss [50]. In an animal model, TNF-α induced hearing loss and increased neuroinflammatory signaling within the cochlea, which could be prevented by TNF-α inhibitors [21,51]. Moreover, a recent in vitro study showed that stress signaling molecules, such as transient receptor potential cation channel subfamily V member 1 (TRPV1), NADPH oxidase 3 (NOX3), TNF-α, and cyclooxygenase 2 (COX2), were upregulated as part of a common mechanism underlying hair cell damage in the inner ear; TNF-α inhibition suppressed this mechanism [52]. So far, the exact effector mechanisms underlying sudden and autoimmune hearing loss are unknown, but a recent TNF-α-related study raised the possibility that a potential connection could exist between ROS and these types of hearing losses.

## 2. Antioxidant Therapies for ROS-Induced Inner Ear Damage 

Studies focusing on antioxidant therapies in ROS-related hearing loss have been reported [53,54]. Conversely, several experiments have shown the preventive effects of antioxidants on age-related, noise-induced, and ototoxicity-induced hearing loss [55,56].

ROS-related damage in the inner ear can be prevented by reducing ROS generation or enhancing the antioxidant system. Specifically, the administration of exogenous antioxidants, the upregulation of endogenous antioxidant production, and the promotion of the ROS scavenger system can prevent inner ear damage [15].

Previous studies have identified a variety of agents that could protect the inner ear from oxidative stress, including *N*-acetylcysteine (NAC), sodium thiosulfate (STS), amifostine, d-methionine, vitamin E, flunarizine, lipoic acid, and ebselen [57,58]. Antioxidants used for protection against or rescue of hearing loss are summarized in Table 1.

### 2.1. N-Acetylcysteine (NAC)

NAC is an acetylated l-cysteine that acts as an antioxidant in two ways. As a thiol, NAC can act directly as an antioxidant. Moreover, as an l-cysteine precursor, NAC stimulates the endogenous antioxidant system [89]. Previous reports have shown that NAC reduced the ototoxic effects of noise exposure in both animal models and humans [59,60,61,62,63,64,65]. For example, Fetoni et al. found that guinea pigs treated with NAC for three days showed a similar temporary threshold shift, and better recovery of compound action potential thresholds with respect to untreated animals [59]. Moreover, treated animals displayed significantly reduced permanent threshold shift and hair cell loss as compared with the control animals. Furthermore, in a study by Bielefeld et al. [60], chinchillas were treated with three different dosages of NAC, i.e., 325, 100, and 50 mg/kg, or with saline as a control, and were exposed to the following three kinds of noise: high-kurtosis (2 h, 108 dB equivalent noise levels (L_eq_)), impulse (75 pairs of 155 dB peak sound pressure level (pSPL) impulses), or continuous (4 kHz octave band, 105 dB sound pressure level (SPL) for 6 h). The protective effects of NAC against high-kurtosis noise were evident even at low doses. In another study by Lorito et al. [61], rats were treated with 375 mg/kg NAC in three different schemes as follows: The first group of rats received four injections for 48 h (pre- and post-noise exposure), the second group was injected prior to exposure, and the third group was injected 24 h after noise exposure, while some non-treated rats served as the controls. The various NAC administration schemes could protect hair cells (as shown by distortion product otoacoustic emissions) or neural fibers (as shown by auditory brainstem responses), but no scheme provided full recovery of cochlear function and neural fibers, at seven days post exposure. The otoprotective property of NAC is thought to be controlled by complicated dynamics. Therefore, the effect of NAC as an otoprotectant exhibits considerable variability due to complex interactions, and differences in dosage and administration timing in different experimental setups.

Some investigators have examined the effects of concomitant administration of other antioxidants together with NAC. Indeed, concomitant administration of antioxidants can produce higher antioxidative effects at lower dosages. For instance, Clifford et al. [62] designated four groups of chinchillas as follows: (1) a group receiving a combination of d-methionine and *N*-acetyl-l-cysteine (12.5 mg/kg each), (2) a group receiving 12.5 mg/kg of d-methionine, (3) a group receiving 12.5 mg/kg of *N*-acetyl-l-cysteine, and (4) saline-treated controls. The chinchillas were exposed to 6 h of continuous 105 ± 0.5 dB SPL octave-band noise centered at 4 kHz. Notably, the combination of d-methionine and NAC led to a significant reduction of inner ear damage at most frequencies. Conversely, the d-methionine-only treated group showed significant recovery of hearing only in the middle frequencies. Another study investigated the effect of intraperitoneal injection of 325 mg/kg NAC combined with 25, 50, or 75 mg/kg sodium salicylate or saline as the control in the recovery from a 6 h exposure to a 105 dB SPL octave-band noise centered at 4 kHz [63]. Intriguingly, the best hearing preservation was observed in the group injected with 325 mg/kg NAC plus 50 mg/kg sodium salicylate. Moreover, substantial reduction of outer hair cell loss was detected in the groups treated with 325 mg/kg NAC plus 50 or 75 mg/kg (but not 25 mg/kg) sodium salicylate. The authors concluded that combinatorial therapy with NAC and sodium salicylate was effective for the prevention of noise-induced damage, despite the relatively narrow therapeutic dosing window of sodium salicylate.

Two randomized clinical trials have assessed the effect of NAC in textile workers [64] and the military population [65]. Textile workers receiving 1200 mg/day of NAC showed reduced noise-induced temporary threshold shift at 4, 6, and 16 kHz [64]. Army members (*n* = 566) were administered NAC or a placebo during weapon training [65]; although no significant differences were observed between groups regarding threshold shift rates, post-hoc analysis highlighted significant differences in threshold shift rates when handedness was considered. In addition, NAC oral administration for 14 days was found to alleviate noise-induced temporary threshold shift [66].

A recent meta-analysis also reported the otoprotective effects of NAC (when administered for more than six weeks) in patients receiving aminoglycoside antibiotics [67]. However, additional studies are needed to clarify the NAC dose response, also considering other confounding factors. NAC has also been shown to play a protective role against cisplatin-induced ototoxicity and noise-induced hearing loss in animal models [36,68]. For instance, guinea pigs treated with transtympanic 2% NAC diluted in normal saline exhibited significant preservation of distortion product otoacoustic emissions in the model of cisplatin ototoxicity [68].

### 2.2. Sodium Thiosulfate (STS)

STS quenches ROS and preserves the activity of antioxidant enzymes [90]. The otoprotective effect of STS has mainly been investigated in cisplatin-induced inner ear damage. However, the disadvantage of the concurrent administration of STS and cisplatin is that STS could compromise the antitumor effect of cisplatin. Nevertheless, the administration of STS 4–6 h after cisplatin treatment could alleviate hearing loss without interfering with the antineoplastic activity of cisplatin [69]. In a randomized controlled trial of pediatric patients treated with cisplatin for hepatoblastoma, a total of 109 children were randomly assigned to groups receiving cisplatin plus STS (57 children) or cisplatin alone (52 children) [70]. STS was administered intravenously over a 15 min period, 6 h after the discontinuation of cisplatin, at a dose of 20 g/m^2^. Because a significantly lower incidence of hearing loss was observed in the cisplatin plus STS-treated group, the authors indicated STS as a potential otoprotective agent.

### 2.3. d-Methionine

d-methionine, the enantiomer of the amino acid l-methionine, is another potent antioxidant that has been considered in preclinical studies [73,91]. d-methionine can directly function as an antioxidant and as an adjuvant of the endogenous antioxidant system [57]. In fact, d-methionine increases intracellular GSH levels [92], specifically those of mitochondrial GSH [93], thereby preserving or improving the ratio of reduced to oxidized GSH in the cochlea [94]. Moreover, d-methionine, administered before or after noise exposure, could rescue noise-induced hearing loss in mice by protecting the cochlear morphology, inhibiting apoptosis, and maintaining the expression levels of connexin 26 and 30 [71]. Moreover, a dose-dependent effect of d-methionine could be observed in the rescue of noise-induced permanent threshold shift [72]. In fact, when guinea pigs were exposed to continuous broadband white noise at 105 dB SPL for 6 h, and then treated five times at 12 h intervals with 200, 400, or 600 mg/kg d-methionine or sterile 0.9% saline, the rescue of noise-induced permanent threshold shift depended on the dose of administered d-methionine, with 200 mg/kg not significantly reducing the mean permanent threshold shift but 600 mg/kg mediating efficient rescue. Similarly, noise-induced decreases in the activities of the Na^+^, K^+^-ATPase, and Ca^2+^-ATPase, as well as in mean lipid peroxidation and nitric oxide levels, were also recovered in a d-methionine dose-dependent manner.

The use of d-methionine, combined with radiation and chemotherapy, protected the hearing threshold at frequencies of 10 kHz and higher [73]. In fact, Wistar rats treated intraperitoneally with 300 mg/kg d-methionine for 30 min before cisplatin infusion, or orally treated with 1000 mg/kg (200 mg/mL) d-methionine delivered by gavage 2 h before infusion of cisplatin, were found to be protected from cisplatin-induced auditory brainstem response threshold shift at all tested frequencies (4, 8, 14, 20, and 30 kHz).

### 2.4. Alpha-Lipoic Acid

Alpha-lipoic acid also contains thiol groups and has been shown to protect the cochlea from cisplatin-induced ototoxicity and noise in animal models [74,76]. Nevertheless, pretreatment with α-lipoic acid (at 100 mg/kg by intraperitoneal administration for two days before cisplatin administration) almost completely protected hearing ability (5–10 dB change in auditory brainstem response threshold) [74]. In the post-treatment group (injected with cisplatin first, and then receiving α-lipoic acid at 100 mg/kg for the next two days), alleviation of hearing loss was also observed, although a small difference in auditory brainstem response threshold changes was observed between the pre- and post-treatment groups. Alpha-lipoic acid exerted a similar protective effect on spiral ganglion cells in both the pre- and post-treatment groups. However, its effect in clinical studies is debatable.

In another animal study of kanamycin-induced ototoxicity, kanamycin-triggered increased auditory brainstem response threshold shifts in BALB/c mice were significantly inhibited by subcutaneous injection of α-lipoic acid. Immunohistochemical staining and Western blot analysis of mouse cochleae showed decreased expression of phosphorylated p38 mitogen-activated protein kinase (MAPK) and phosphorylated c-Jun N-terminal kinase (JNK) [75].

Furthermore, in an age-related hearing loss A/J mouse model, measurement of the auditory brainstem response thresholds revealed greater hearing loss attenuation in the group treated with 50 μg/g α-lipoic acid than in the controls [76]. Additionally, α-lipoic acid-dependent preservation of outer hair cells, spiral ganglion neurons, and stria vascularis was observed in the cochleae of A/J mice.

Quaranta et al. conducted a preliminary clinical trial on 30 healthy subjects and measured a temporary threshold shift of hearing of 6 kHz 2 min after the end of noise exposure (90 dB hearing loss (HL) 3 kHz pure tone for 10 min) [77]. Interestingly, subjects that had been orally treated with 600 mg of α-lipoic acid for 10 days showed lower threshold shifts as compared with subjects that had not received α-lipoic acid or orally ingested 600 mg of α-lipoic acid once, 1 h before exposure. The authors also observed similar results in transient evoked otoacoustic emission. From these results, the authors suggested the otoprotective effect of α-lipoic acid.

### 2.5. Amifostine

Amifostine is a prodrug that provides selective protection for healthy tissues. Its active metabolite, WR-1065, protects the cochlea by scavenging ROS. In animal models, systemic administration of amifostine protected outer hair cells of the cochlea [78,95] and their functioning (measured in terms of distortion product otoacoustic emissions) from cisplatin-induced ototoxicity [78]. However, clinical studies have failed to reach a consistent conclusion with regard to the effects of amifostine [79,80,81]. In a large trial with 379 patients affected by medulloblastoma, it was evaluated whether treatment with 600 mg/m^2^ amifostine immediately before or 3 h after each cisplatin infusion could alleviate hearing loss [80]. Although this study was not randomized, it was found that amifostine was associated with protection from serious hearing loss (defined as hearing loss requiring a hearing aid or deafness). Another study involving 242 ovarian cancer patients receiving cyclophosphamide and cisplatin observed a 43% reduction in the incidence of ototoxicity (defined as clinical hearing loss or tinnitus, that required dose reduction or discontinuation of cisplatin treatment); however, the difference with the control group did not reach statistical significance [81].

### 2.6. Ebselen

Ebselen is a selenium-containing compound. It serves as an antioxidant, scavenges ROS by mimicking the GSH peroxidase, is an anti-inflammatory agent, and an activator of nuclear factor, erythroid 2-like 2 (NRF2) [96,97]. In animal models of breast and ovarian cancer, ebselen combined with allopurinol provided protection against cisplatin-induced ototoxicity to a certain extent [82]. Notably, the combination of these two compounds did not interfere with but rather enhanced the antitumor activity of cisplatin.

Guinea pigs receiving an oral dose of 10 mg/kg ebselen, 1 h before exposure to 115 dB SPL 4 kHz octave-band noise for 3 h, were protected from temporary threshold shift [83]. In the control group, swelling of the afferent dendrites beneath the inner hair cells was observed immediately after noise exposure, whereas ebselen significantly reduced these pathological damages. Moreover, in a randomized, double-blind, placebo-controlled, phase 2 trial to validate the safety and efficacy of ebselen for the prevention of noise-induced hearing loss [84], 83 participants were allocated to receive 200, 400, or 600 mg ebselen or placebo before a calibrated sound challenge (4 h of pre-recorded music delivered by insert earphones). In this study, the authors suggested that treatment with ebselen, at a dose of 400 mg twice daily, was safe and effective in preventing noise-induced temporary threshold shifts. As an organo-selenium compound, ebselen mimics GSH peroxidases and shows considerable potential for preventing noise-induced hearing loss in the future [84].

### 2.7. Flunarizine

Flunarizine decreases proinflammatory cytokine secretion by downregulating the expression of nuclear factor-kappa B (NF-κB) and MAPK through the activation of NRF2 [98]. However, the mechanism underlying flunarizine-mediated cochlear protection is still unclear, and its efficacy has only been shown in vitro [99].

### 2.8. Other Antioxidants

Other antioxidants that may potentially play a protective role against cochlear oxidative stress include ginseng [64], coenzyme Q10 (CoQ10) [88], and vitamins such as vitamin A [100], C [101], E [102], and B12 [103]. Several studies have shown the protective effects of combined antioxidant treatments in animals [104], but the outcome of such treatments in humans still needs to be investigated.

Korean red ginseng has anti-ROS and anti-apoptotic properties, and therefore may play a role in the prevention of noise-induced hearing loss. For example, Kang et al. fed Korean red ginseng (200 mg/kg) to BALB/c mice for three days after exposure to 110 dB noise for 3 h to induce temporary threshold shift [85]. These authors observed a faster recovery of hearing loss in mice fed with Korean red ginseng 1 h and one day after noise exposure as compared with mice fed with Korean red ginseng three days after noise exposure. Moreover, 8-oxoguanine, a known indicator of ROS production after noise exposure, was not observed in the stria vascularis of mice fed with Korean red ginseng as compared with the control group. Additionally, Choung et al. treated rats with Korean red ginseng (500 mg/kg) for 12 days prior to intraperitoneal injection of gentamycin [86]. These authors detected significantly improved hearing in these rats as compared with the group that did not receive Korean red ginseng.

CoQ10 is a component of the mitochondrial respiratory chain. It inhibits mitochondrial lipid peroxidation, induces ATP production, and is involved in ROS scavenging and prevention of oxidative stress-induced apoptosis. Fetoni et al. injected guinea pigs intraperitoneally with water-soluble CoQ10 at a dose of 100 mg/kg and subjected them to 120 dB SPL pure tone sound at a frequency of 6 kHz for 60 min [87]. CoQ10 significantly reduced hearing loss 21 days after noise exposure, as demonstrated by auditory brainstem responses and decreased signs of apoptosis by active caspase 3 staining and TUNEL assay. Inner ear hair cell loss was also prevented. Similarly, in a clinical study, Staffa et al. found a faster recovery of hearing after noise exposure in subjects that ingested CoQ10 as a food supplement for 30 days [88].

Mitoquinone, a mitochondria-targeted derivative of the antioxidant ubiquinone, was also suggested as an otoprotective agent [105]. Nevertheless, when amikacin-treated guinea pigs received oral mitoquinone (supplemented at 30 mg/L in drinking water) or were injected subcutaneously with mitoquinone at 3 to 5 mg/kg, limited protection was observed against amikacin-induced hearing loss at 24 kHz.

Aspirin reportedly exhibits a preventive effect on cisplatin-induced ototoxicity [106]. Similarly, salicylate is a clinically promising antidote against it [37]. Aspirin might also exert a similar physiological antioxidant effect to that of salicylate, as it can be converted into this compound. A meta-analysis also showed the potential of aspirin administration for the prevention of ototoxicity based on the paradoxical effect of aspirin [107]. In contrast, aspirin failed to show a significant protective effect against ototoxicity in a multi-center randomized trial [108]. Therefore, further research is required to verify the effects of aspirin on ototoxicity.

## 3. Antioxidant Therapeutic Mechanisms in the Inner Ear

Since excessive free radical buildup in the inner ear is clearly a key factor in the pathogenesis of various types of hearing loss, increasing cochlear antioxidant supplies could substantially prevent hair cell damage and hearing loss. Exogenous antioxidant agents, such as various free radical scavengers including GSH [16], ebselen [109], and d-methionine [94] have shown to reduce sensory cell death and noise-induced hearing loss in animal models. In the case of vitamins, a combined treatment using magnesium and vitamins A, C, and E has reportedly been more efficient than the independent use of any agent, probably due to distinct effector mechanisms in each case, leading to synergistic effects [104]. Furthermore, dietary supplementation of antioxidants, such as all-trans retinoic acid (ATRA) and α-lipoic acid, attenuated noise exposure-induced apoptosis [110] and the accumulation of oxidative DNA lesions mediated by early-onset age-related hearing impairment [111], respectively. Post-exposure administration of ATRA, in particular, was found to recover hearing threshold shifts and reduce hair cell loss [100]. Moreover, the therapeutic effects of two pharmacological agents, NAC and acetyl-L-carnitine (ACAR), after acoustic trauma, reportedly led to the recovery of permanent threshold shifts and ameliorated hair cell loss [112]. NAC functions as a direct H_2_O_2_ and HO^−^ scavenger and is a major contributor to cellular GSH synthesis. ACAR improves the maintenance of mitochondrial membrane energetics and integrity. The endogenous antioxidant enzymatic system has evolved to control the detrimental effects of free radicals in the ear, including increased levels of antioxidant enzymes, such as copper/zinc and manganese superoxide dismutases (SOD1 and SOD2, respectively), catalase, GSH-related enzymes (glutathione peroxidases (GPxs), glutathione transferases (GSTs), and glutathione reductase), and peroxiredoxins (PRDXs). SODs can dismutate two O_2_^−^ anions into H_2_O_2_ and molecular oxygen. In addition to differential requirements for metal ions as cofactors, SODs have distinct subcellular localizations; SOD1 is localized in the cytoplasm and extracellularly, while SOD2 is localized in the mitochondria. Spontaneous and enzymatically catalyzed reactions, including plasma membrane-associated NADPH oxidases, the mitochondrial electron transport chain, the cytosolic xanthine oxidase, and cytochrome p450 monooxygenases mainly acting in the endoplasmic reticulum, can give rise to O_2_^.^^−^. Therefore, SODs are regarded as the first line of endogenous antioxidant enzymes to convert toxic ROS to less harmful H_2_O_2_ [113].

**Figure 1:**
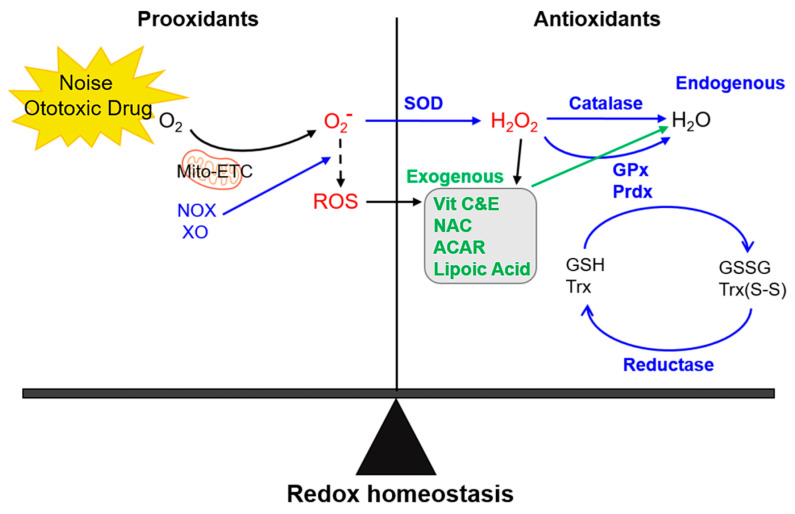
Mechanism of redox homeostasis. The balance between intracellular reactive oxygen species (ROS) generation and exogenous and endogenous antioxidant effector mechanisms maintains acoustic integrity. The mitochondrial electron chain reactions, as well as NOX and XO activation, represent major sources of intracellular ROS, such as O_2_^−^, HO^−^, and H_2_O_2_. The blue letters and arrows indicate endogenous pathways and the green letters and arrows indicate exogenous pathways. Mito-ETC, mitochondrial e^−^ transport chain reactions; NOX, NADPH oxidase; XO, xanthine oxidase; NAC, *N*-acetyl cysteine; ACAR, acetyl-L-carnitine; SOD, superoxide dismutases; GPx, glutathione peroxidases; PRDXs, peroxiredoxins; TRX, thioredoxins.

The tetrameric enzyme catalase is, then, responsible for detoxification through the breakdown of two hydrogen peroxide molecules into one molecule of oxygen and two molecules of water. According to the differences in their sequences and structures, catalases are divided in three types. Monofunctional heme-containing catalases are the most widespread type, present in all aerobic organisms. Bifunctional catalases-peroxidases carrying a heme group belong to the second type, which is relatively less abundant in nature. The third type consists of Mn-containing catalases that lack the heme group [114]. GPxs are another group of enzymes capable of reducing H_2_O_2_ or organic hydroperoxides to water or corresponding alcohols using reduced GSH as an electron donor (H_2_O_2_ + 2GSH = GS-SG → 2H_2_O). GPxs display a selenium dependent GSH peroxidase activity, relying on a selenocysteine residue encoded by an opal TGA codon. In mammalian tissues, at least four types of selenium dependent GPx isozymes have been identified; a classical GPx (GPx1), a gastrointestinal GPx (GPx2), a plasma GPx (GPx3), and a phospholipid GPx (GPx4). GPx1–3 act as homotetramers, while GPx4 functions as a monomer. These isozymes have distinct subcellular locations, such as cytosol, nucleus, and mitochondria for GPx1; cytosol and nucleus for GPx2; cytosol and extracellular space for GPx3; and nucleus, cytosol, mitochondria, and membranes for GPx4 [115]. PRDXs belong to the superfamily of nonheme and non-selenium peroxidases, which catalyze the thiol-dependent reduction of a wide range of peroxides. They are ubiquitously distributed throughout all phyla and classified as 1- or 2-Cys subgroups based on their number of conserved cysteine residue(s) per monomer involved in the catalytic mechanism. Among the six mammalian PRDXs, PRDX1–4 function as dimers and use the N-terminal Cys (peroxidatic Cys) of one monomer to reduce hydroperoxides; the resultant oxidized (sulfenic, -SOH) Cys interacts with the C-terminal Cys of the other monomer (the resolving Cys) to generate a protein disulfide. PRDX5 is classified as an atypical 2-Cys PRDX with both the peroxidatic and the resolving Cys on the same monomer, forming an internal disulfide during the catalytic cycle. These 2-Cys PRDXs utilize thioredoxin as an electron donor to reduce disulfides. PRDX6 is the sole 1-Cys enzyme displaying both peroxidase and phospholipase A2 activities. The reduction of hydroperoxides by PRDX6 occurs through oxidation of its single conserved Cys at position 47. Due to the absence of a second Cys, as in 2-Cys PRDXs, the resolution of the sulfenic state requires the formation of heterodimers between PRDX6 and πGST in the presence of GSH as a physiological reductant. Another distinct characteristic of PRDX6 is its ability to bind and reduce phospholipid hydroperoxides [116]. These antioxidant enzymes maintain the redox homeostasis, thereby contributing to cellular stability and viability in the ear [117,118,119]. Free radical generation during noise exposure and the effector mechanisms of exogenous and endogenous antioxidants are illustrated in Figure 1.

## 4. Preconditioning Effects in the Inner Ear

The preconditioning effect is an interesting phenomenon observed during hair loss-prevention studies. Preconditioning refers to the process of pre-inducing a preventative pathway by subtle stimuli that do not cause permanent damage in the inner ear. After preconditioning, the inner ear is more resistant to the actual major stimuli that are supposed to cause permanent damage in the inner ear. In other words, preconditioning refers to providing the cells with continuous weak stress, and thereby allowing them to develop the ability to protect themselves against significant stress stimuli. In the inner ear, several types of preconditioning have been studied, i.e., hyperthermia [120], restraint [121], hypoxia [122,123], and sound [124,125,126].

The effector mechanisms of such preconditioning stimuli have been identified as increased levels of heat shock proteins [120], glucocorticoids [121], and antioxidants, including GSH reductase, glutamyl cysteine synthetase, and catalase [127], in the body. These heightened signals inhibit the critical damage leading to noise-induced hearing loss, which culminates in apoptosis. Preconditioning through sound stimulation might reduce cytochrome C expression, while increasing the expression levels of the cell death inhibitory protein BCL2 and reducing DNA fragmentation, thereby preventing cell death [128]. Therefore, a preconditioning strategy might activate the intracellular antioxidant system, possibly conferring resistance to oxidative damage.

### 4.1. Preconditioning by Sound in Noise-Induced Hearing Loss

Sound conditioning is a strategy to protect hearing damage by creating resistance to harmful intense noise stimuli through pretreatment with low-level noise stimuli [124]. The mechanisms of sound conditioning-mediated resistance in the inner ear might consist of the activation of the antioxidant system [125], an increase of tyrosine hydroxylase levels in the lateral efferent system [129], and increased production of glucocorticoids [120] and other chemicals. The increase in glucocorticoid levels is caused by the activation of the hypothalamic-pituitary-adrenal axis, while downregulated expression of the glucocorticoid receptor occurs upon exposure to noise. Conversely, sound conditioning triggers upregulated expression of the glucocorticoid receptor in the cochlea through the activation of steroid receptor co-activator-1 (SRC-1). Through these signaling mechanisms, sound conditioning suppresses noise-induced hearing loss [130]. Thus, the levels of glucocorticoids and the expression of their receptor play an important role in preventing hearing loss.

### 4.2. Preconditioning by Hyperthermia and Restraint

The preconditioning effect is not only caused by sound (sound conditioning). It has also been reported that the incidence of noise-induced hearing loss was reduced when systemic stress was increased [131]. Both hyperthermia and restraint also induce an increase in the levels of steroid hormones in the body. In fact, Yoshida et al. stimulated mice with heat stress, for approximately 15 min, with rising rectal temperature to 41.5 °C on a heating pad [120]. When mice were exposed to noise after heat stress, these authors observed that the occurrence of hearing loss was decreased as compared with control mice without heat stress. However, the protective effect of heat stress against noise-induced hearing loss gradually decreased in a time-dependent manner and resulted almost null after 24 h. Moreover, these authors observed an increase in the levels of *HSP70* mRNA in cochlea, suggesting that the corresponding protein is involved in the protective mechanism of heat stress.

After restraining mice in plastic tubes for 12 h, Wang et al. exposed them to a noise level that caused hearing loss [121]. The restraint itself did not affect the hearing ability of mice, but noise-induced hearing impairment was reduced in restrained mice with respect to the control group. Restraining showed good protective effects against hearing loss 2 h after a restraint of 12 h, but the protective effect decreased with time. Two hours after the restraint, the corticosterone level in mouse serum increased, but gradually decreased thereafter. Therefore, it is likely that the protective effect of restraining is correlated with increasing glucocorticoid levels in the body. Similarly, the circadian increase of steroid levels in the body might induce the same effector mechanisms as those associated with steroid-dependent protection against noise stimulation [132,133]. Supplementation of prednisolone was shown to inhibit the noise-induced temporary threshold shift of hearing [132]. Moreover, serum aldosterone levels reportedly decreased upon noise exposure [134].

### 4.3. Hypoxic Preconditioning

**Figure 2:**
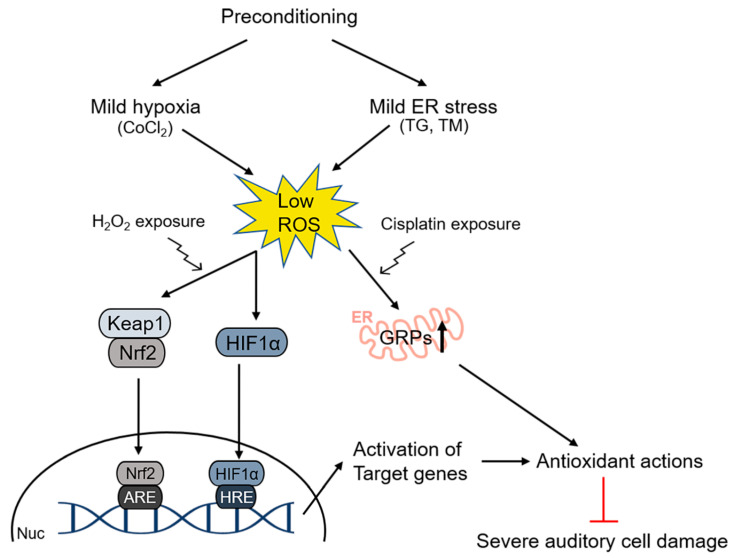
Example of preconditioning effects on ototoxicity induced by auditory hair cell exposure to H_2_O_2_ and cisplatin. Prior to the exposure to half-maximal cytotoxic doses of H_2_O_2_ or cisplatin, HEI-OC1 cells were pretreated with CoCl_2_, thapsigargin, or tunicamycin. These pretreatments resulted in reduced levels of ROS, thereby protecting cells from apoptotic cell death caused by subsequent oxidative stress. Red lines indicate the blocking of the cell damage by antioxidants. ER, endoplasmic reticulum; TG, thapsigargin; TM, tunicamycin; GRPs, glucose-related proteins; Nrf2, nuclear factor erythroid-2-related factor 2; HIF1α, hypoxia-inducible factor 1-alpha; ARE, antioxidant response element; HRE, hypoxia response element; Nuc, nucleus.

In addition, hypoxic preconditioning conferred significant protection against broadband noise exposure via the activation of hypoxia-inducible factor 1 subunit alpha (HIF-1α) within the organ of Corti [123]. Under normoxic conditions, HIF-1α is hydroxylated and degraded [135]. However, under hypoxic conditions, HIF-1α is not hydroxylated and accumulates in the tissue. As cobalt ions can induce tissue hypoxia, HIF-1α expression could be induced by cobalt chloride (CoCl_2_). In CoCl_2_-treated mice, noise exposure, which was supposed to induce a permanent threshold shift of hearing, could not induce permanent hearing loss [122]. In contrast, YC-1, a HIF-1α inhibitor, attenuated this protective role of CoCl_2_. A subsequent study revealed that CoCl_2_ pretreatment protected auditory hair cells (HEI-OC1) from H_2_O_2_-mediated cytotoxicity via the activation of the redox-sensitive transcription factors HIF-1α and NRF2, as well as their target gene *PRDX6*, indicating the protective roles of antioxidant enzymes against noise-triggered oxidative damage [136]. The involvement of oxidative stress in hearing impairment has also been reported in the case of cisplatin-mediated ototoxicity, where mild endoplasmic reticulum (ER)-related stress-induced upregulated expression of glucose-related protein (GRP) 78 and 94, resulting in the attenuation of intracellular ROS accumulation and cisplatin-triggered caspase-associated apoptotic signaling in HEI-OC1 cells [137], as shown in Figure 2.

### 4.4. Unfolded Protein Response (UPR)

The unfolded protein response (UPR) is responsible for protein quality control through the upregulated expression of ER chaperones, the inhibition of de novo protein synthesis, and the removal of misfolded proteins when overaccumulated in the ER [138,139,140]. However, if the UPR fails to control the homeostasis of cells in response to ER stress, apoptosis is induced. The fact that the UPR involves both genes responsible for the two conflicting functions of cell protection, and apoptosis has raised questions about how the balance between them is maintained. Accordingly, several studies have reported the mechanisms underlying the UPR [139].

In mammalian cells, the UPR is induced by IRE1 [141], PERK [142], and ATF6 [143], which are the three types of ER stress sensors present in the ER membrane. The kinase/RNase function of IRE1 is activated by ER stress to induce splicing of *XBP1* mRNA, leading to the production of an active XBP1 transcriptional factor [144]. XBP1 enhances the folding capacity of proteins in the ER by inducing the expression of ER chaperones such as BiP. In the case of PERK, this sensor induces phosphorylation of the translation initiator factor eIF2α to reduce protein synthesis. Moreover, PERK induces the expression of genes involved in UPR by promoting the expression of certain transcriptional factors, such as ATF4 [139].

Because PERK can also induce genes that cause apoptosis, such as *CHOP* and genes coding for b-ZIP transcription factors, PERK triggers apoptosis in excessive ER-stress environments. Moreover, ATF6, as an active transcriptional factor, induces the expression of genes involved in UPR by releasing its cytoplasmic domain via proteolytic cleavage. A study of the association between hearing loss and ER stress showed that, in cells carrying mutated *GJB2*, the corresponding mutant protein accumulated in the ER and failed to move to the plasma membrane, causing hearing loss [145]. Notably, *GJB2* mutations are found in patients with nonsyndromic hearing loss. Additionally, in mice that suffered from hearing loss by intracochlear injection of chemicals inducing mitochondrial dysfunction, CHOP, one of the ER stress sensors, was expressed in the lateral wall of the cochlea before the occurrence of apoptosis [146]. These findings indicated that damage in the inner ear and ER stress were related.

When weak ER stress was preconditioned using ER stress inducers in a renal epithelial cell line, cellular injury induced by H_2_O_2_ decreased [147]. Similar protective effects were observed in cells damaged by cisplatin and gentamicin [148]. Furthermore, when anti-Thy1 nephritis was induced in rats preconditioned by ER stress, tissue damage was significantly reduced with respect to rats that did not receive preconditioning [149]. Similarly, oxidative stress could be inhibited by preconditioning with ER stress through the upregulation of extracellular signal-regulated protein kinase (ERK) and the downregulation of JNK.

Finally, when ischemic preconditioning was induced, AKT phosphorylation and the levels of HIF-1α and GRP78 were increased, while the levels of PERK and ATF4 were reduced, resulting in protection from renal ischemia-reperfusion injury [150]. Moreover, ATF6α increases protein folding and secretion during ER stress. Increased ATF6α expression can restore normal cellular functioning during acute stress and maintain tolerance during chronic stress [151]. In addition, preconditioning by hyperthermia activated the adaptive pathway through UPR and prevented cellular damage by oxidative stress [152]. Accordingly, mild ER stress can activate the adaptive pathway of UPR and may be an important preconditioning strategy.

## 5. Conclusions

As ROS-related tissue damage is the major pathway underlying hearing loss; several pieces of evidence could potentially prove the efficiency of antioxidant therapies against noise-, aging-, and ototoxicity-induced hearing loss. Several compounds have shown a protective or rescuing effect against hearing loss in clinical studies, namely NAC (combined with salicylate), alpha-lipoic acid, amifostine, ebselen, and coenzyme Q10. However, further well-designed clinical studies should be conducted to confirm the protective effects of these compounds.

## Figures and Tables

**Table 1:** List of antioxidants used for protection of hearing loss.

Name	Study Subjects		Condition	Outcome Regarding Hearing Loss	Reference
	Animal Model	Clinical Study			
*N*-acetyl cysteine	Guinea pig		Noise	Protection	[59]
	Chinchilla		Noise	Protection	[60]
	Rat		Noise	Protection	[61]
Combined with d-methionine	Chinchilla		Noise	Protection	[62]
Combined with salicylate	Chinchilla		Noise	Protection	[63]
		48 textile workers, RCT, phase 2	Noise	Protection	[64]
		634 military population during weapon training, phase 2	Noise	Partial effect (post-hoc analysis, handedness)	[65]
		31 normal-hearing participants, phase 2	Noise	No effect in this study setting	[66]
		Meta-analysis, 3 studies, 146 patients with end-stage renal disease	Aminoglycoside	Protection	[67]
	Guinea pig		Cisplatin	Protection	[68]
Sodium thiosulfate	Rat		Cisplatin	Rescue	[69]
		109 pediatric patients with hepatoblastoma, RCT, phase 3	Cisplatin	Rescue	[70]
d-methionine	Mouse		Noise	Rescue	[71]
	Guinea pig		Noise	Rescue	[72]
	Rat		Noise	Protection	[73]
Lipoic acid	Mouse		Cisplatin	Protection, rescue	[74]
	Mouse		Kanamycin	Protection	[75]
	Mouse		Aging	Protection	[76]
		30 normal-hearing participants	Noise	Protection	[77]
Amifostine	Guinea pig		Cisplatin	Protection	[78]
		9 pediatric patients with medulloblastoma	Cisplatin	No effect in this study setting	[79]
		379 children with medulloblastoma, not randomized	Cisplatin	Protection from serious hearing loss in average-risk patients	[80]
		242 ovarian cancer patients, RCT, phase 3	Cisplatin	No effect in this study setting	[81]
Ebselen	Rat		Cisplatin	Protection	[82]
	Guinea pig		Noise	Protection	[83]
		83 normal-hearing participants, RCT, phase 2 trial	Noise	Protection	[84]
Korean red ginseng	Mouse		Noise	Rescue	[85]
	Rat		Gentamicin	Protection	[86]
Coenzyme Q10	Guinea pig		Noise	Protection	[87]
		30 normal-hearing participants	Noise	Protection	[88]

RCT, randomized clinical trial.
